# Relationship between Dietary Patterns and Cardiovascular Disease Risk in Korean Older Adults

**DOI:** 10.3390/ijerph18073703

**Published:** 2021-04-01

**Authors:** Ae-Rim Seo, Tae-Yoon Hwang

**Affiliations:** 1 Department of Preventive Medicine and Institute of Health Science, Gyeongsang National University College of Medicine, Jinju 52727, Korea; sarim2101@naver.com; 2 Department of Public Health, Yeungnam University Graduate School, Gyeongsan 38541, Korea; 3 Department of Preventive Medicine and Public Health, Yeungnam University College of Medicine, Deagu 42415, Korea

**Keywords:** diet, heart disease, risk, aged

## Abstract

Objectives: The purpose of this study was to assess the relationship between dietary patterns and the 10-year risk of cardiovascular disease (CVD) in the elderly population in Korea. Methods: Cluster analysis was conducted on the data of 1687 elderly participants (797 men and 890 women) aged ≥65 years from the 2014–2016 Korea National Health and Nutrition Examination Survey (KNHANES), using a 24-h dietary recall survey to assess dietary patterns. Dietary patterns were classified into clusters 1 (typical Korean diet), 2 (high carbohydrate diet), and 3 (healthy diet). The 10-year risk of CVD was calculated based on age, total and HDL-cholesterol levels, systolic blood pressure level, antihypertensive medication use, smoking status, and presence of diabetes. A complex sample general linear model was applied to determine the association between dietary patterns and the 10-year risk of CVD. Results: In total, 275 (33.7%), 141 (17.9%), and 381 (48.3%) men, and 207 (22.6%), 276 (30.9%), and 407(46.6%) women were included in clusters 1, 2, and 3, respectively. The 10-year risk of CVD was lower in men in cluster 3 (healthy diet) than in those in cluster 1 (typical Korean diet) (t = 2.092, *p* = 0.037). Additionally, the 10-year risk of CVD was lower in men who performed strength training than in those who did not (t = 3.575, *p* < 0.001). There were no significant differences in women. Conclusions: After adjusting for sociodemographic variables, men who consumed a healthy diet had a lower 10-year risk of CVD than those who consumed a typical Korean diet. When organizing nutrition education programs to improve dietary habits in the elderly, content on diets that consist of various food groups to prevent CVD is required. In particular, it is necessary to develop content that emphasizes the importance of healthy eating habits in men.

## 1. Introduction

The three major causes of death in Korea are cancer, heart disease, and stroke, which account for 45.0% of the total number of deaths [1]. In particular, the mortality rate due to heart disease is 62.4 per 100,000 people, and it increases with age, as observed in 11.2, 27.2, 61.4, and 216.0 per 100,000 people for those in their 40s, 50s, 60s, and 70s, respectively [1]. The proportion of elderly people in South Korea is expected to increase rapidly from 13.8% in 2017 to 20% and 30% in 2025 and 2036, respectively [2]. Additionally, the socioeconomic burden associated with cardiovascular disease (CVD) due to aging is expected to increase further.

Artnian et al. [3] suggested an increase in physical activity level, consumption of a healthy diet, smoking cessation, and weight loss to reduce the risk factors for CVD in adults. The American Heart Association (2019) also emphasized the need for proper weight management and diet, drug therapy, smoking cessation, and constant exercise to prevent atherosclerotic CVD [4].

A study on the relationship between diet and CVD risk reported that the intake of vegetables, fruits, whole grains, and nuts has beneficial effects on cardiovascular health, and that fish consumption reduces the risk of CVD [5]. Recommendations on diet for cardiovascular patients include reduced intake of saturated and trans fatty acid; increased intake of unsaturated fatty acids, complex carbohydrates, and dietary fiber; low sodium intake; and diet for weight management [6].

Several nutrients are ingested together during food intake, and the nutrients interact and metabolize in the body [7]. It is difficult to make definite conclusions on the association between diet and disease from a study on a single nutrient [8]; however, recently, the interest in evaluation using dietary patterns, diet guidelines, and diet quality indices is increasing [9].

The Mediterranean diet, known to be a healthy diet, and dietary approaches to stop hypertension (DASH) were reported to reduce the risk of coronary artery disease [10]. The DASH diet aims to reduce saturated fatty acids and total fat levels by increasing the intake of fruits, vegetables, and fat-free and low-fat milk and dairy products [11].

A Korean study on the relationship between dietary patterns and chronic diseases in adults showed that there was a significant reduction in the risk of metabolic syndrome in the healthy dietary pattern compared to grain and white rice dietary pattern. In this study, subjects were divided into three groups based on dietary pattern: healthy dietary pattern (the highest intakes of vegetables, fruits, legumes, and fish), grain dietary pattern (the highest intakes of grain), and white rice dietary pattern (the highest intakes of white rice) [12]. Another study showed that diets rich in fruits, vegetables, whole grains, legumes, peanuts, and fish lowered the prevalence of metabolic syndrome. This study used a modified Mediterranean diet scoring system (ranged from 0 to 9). The higher the score, the richer the diet in fruits, vegetables, whole grains, legumes, peanuts, and fish. Participants with 5 or higher diet scores had a lower prevalence of metabolic syndrome in overall population [13]. 

Adequate nutrient intake in the elderly is one of the important factors that promote health and improve the quality of life [14,15]. Nutritional imbalance and lack of nutrients in the elderly can cause a severe functional decline, associated with a high risk of disease and mortality [16].

Therefore, this study aimed to assess the dietary patterns of the elderly in Korea and assess the relationship between dietary patterns and the 10-year risk of CVD.

## 2. Subjects and Methods

### 2.1. Study Participants

**Figure 1:**
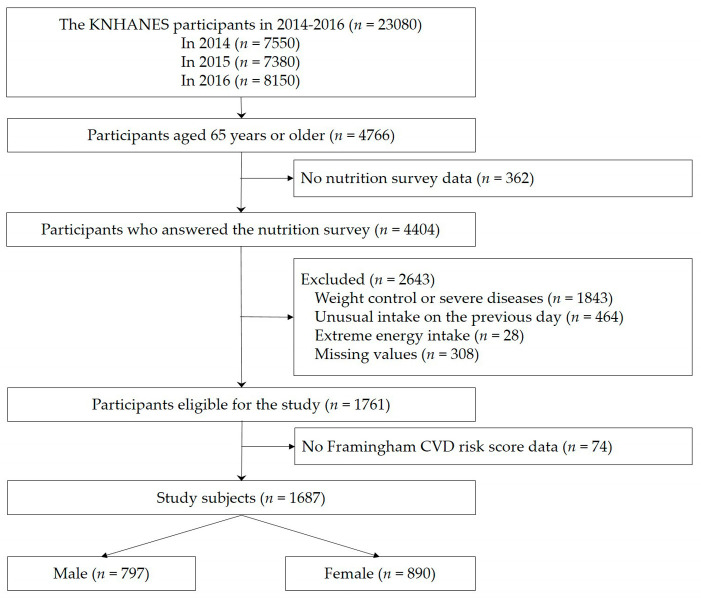
Flowchart of the study subjects.

This study was based on data from the Korea National Health and Nutrition Examination Survey (KNHANES), 2014–2016. The KNHANES includes representative statistical data of the Korean population, and it consists of a health interview survey, health examination, and food intake survey. In total, 4404 participants over 65 years of age were included in this study. The exclusion criteria were as follows: changes in eating habit due to severe diseases or weight control; unusual intake on the previous day; extremely low or high energy intake (under 500 or over 5000 kcal/day); CVD (myocardial infarction and angina), stroke, cancer, or renal failure; and missing data from the questionnaires. Additionally, those without data (age, high-density cholesterol, total cholesterol, systolic blood pressure, hypertension treatment, smoking status, and diabetes) to calculate the 10-year risk of CVD were excluded. Finally, 1687 subjects (797 men and 890 women) were included in the analysis (Figure 1).

This study was approved by the Institutional Review Board of Yeungnam University (approval number: 2 August 2018).

### 2.2. Sociodemographic and Health-Related Variables 

Sociodemographic characteristics included age, educational level, income, and marital status. Age group was classified into 65–74 years and >75 years. Educational level included elementary school, middle school, high school, and university. Income was divided into quartiles (high, upper middle, lower middle, low), and marital status was classified as living with spouse or living alone.

Health-related characteristics included strength training, smoking status, and alcohol consumption. Strength training was assessed as being performed less or more than twice a week. Smoking status was categorized as current smokers and non-smokers (including past smokers). High-risk alcohol consumption was defined as the intake of seven or more drinks per occasion for men and five or more drinks per occasion for women, two or more times within the past 7 days.

In the examination data, body mass index (BMI) and blood pressure were included in the analysis.

### 2.3. Dietary Patterns

The food intake survey included the name of each food, ingredient of each food, and food intake quantity during the entire day, immediately before the survey (24-h dietary recall survey). Their responses were classified into 18 food groups according to the food code system suggested by the KNHANES. Grains and their products accounted for almost half of the daily energy intake, so they were subdivided into white rice, other grains, noodles (including dumplings), flour (including bread), pizza (including hamburger, cereals, and snack), and potatoes. Kimchi (Korean traditional fermented cabbage) was grouped separately from other vegetables because of its higher sodium content and higher intake by Koreans compared to other vegetables. In the analysis, 22 food groups were reclassified [17] (Supplementary Table S1).

Dietary patterns were determined by performing K-means cluster analysis (10 repeated trials). The cluster analysis method involves grouping a set of participants in such a way that participants in the same group are more similar to each other than to those in other clusters [18]. Using cluster analysis, we used the portion size of the food consumed by the 22 reclassified food groups (a portion size of each food intake of the total intake per day) and extracted three dietary patterns [19]. 

The dietary reference intakes for Koreans (KDRIs) in 2015 differed according to sex and age [20], while dietary habits and food preferences differed according to sex [21]. Thus, dietary patterns for both men and women were assessed. Cluster 1 included men and women who consumed a “typical Korean diet” with a lot of white rice and kimchi. Cluster 2 included those who had a “high carbohydrate diet,” where men mainly consumed bread and alcohol, while women mainly consumed hamburgers, snacks, and fruits. Cluster 3 consisted of men and women who consumed a “healthy diet” with a lot of whole grains, vegetables, fish, and dairy products (Supplementary Table S2).

### 2.4. Ten-Year Risk of CVD 

The CVD risk score and 10-year risk of cardiovascular incidents in the participants were calculated using the Framingham criteria [22], which differed depending on the sex; higher scores indicated higher CVD risk. 

The Framingham CVD risk score (FRS) and 10-year risk of cardiovascular incidence were calculated based on age, total and HDL-cholesterol levels, systolic blood pressure level, antihypertensive medication use, smoking status, and presence of diabetes [21]. The cumulative effect of the risk of developing CVDs in the coming 10 years was defined as the 10-year risk of cardiovascular incidents.

In calculating FRS, information on sex, age, smoking status, and hypertension medication use was obtained from the health interview survey data, while that on fasting blood sugar and total and HDL-cholesterol levels was obtained from health examination results. Diabetes was assessed by measuring the fasting blood sugar level in accordance with the 2015 diabetes treatment guidelines by the Korean diabetes association. The average of systolic blood pressure measured 2 or 3 times was used, excluding the first measured blood pressure in the data from the KNHANES, which was measured three times. It was measured after smoking cessation for 30 min; a 6-min break was taken in a sitting position before another measurement.

### 2.5. Statistical Analysis

Statistical analyses were performed using SAS version 9.3 (SAS Institute Inc., Cary, NC, USA) and SPSS version 25.0 (IBM Corp., Armonk, NY, USA). We analyzed the KNHANES data with primary sampling units, strata, and integrated weights because they were collected using a complex sampling design involving cluster and stratified sampling. Complex sample general linear model was applied to determine the association between dietary patterns and a 10-year risk of CVD. Adjusted variables were marital status, educational level, income, strength training status, alcohol consumption, and BMI, which were known to be associated with CVD risk in a previous study [23]. Age, sex, smoking status, hypertension medication use, presence of diabetes, and total and HDL-cholesterol levels were excluded from the analysis as they were included in the formula for calculating the 10-year risk of CVD. All tests were two-tailed, with *p*-value < 0.05 being considered statistically significant. The between-group differences in the mean were assessed by ANOVA followed by Bonferroni’s post hoc test.

## 3. Results

### 3.1. Sociodemographic and Health-Related Variables According to Dietary Patterns

In total, 275 (33.7%), 141 (17.9%), and 381 (48.3%) men and 207 (22.6%), 276 (30.9%), and 407(46.6%) women were included in clusters 1, 2, and 3, respectively

There were significant differences according to age (*p* = 0.005), educational level (*p* < 0.001), income (*p* < 0.001), high-risk alcohol consumption (*p* < 0.01), and diagnosis of dyslipidemia (*p* = 0.038) among men. In women, there were significant differences according to age (*p* < 0.001), educational level (*p* < 0.001), strength training (*p* = 0.038), and diagnosis of dyslipidemia (*p* = 0.001) (Table 1).

### 3.2. Ten-Year Risk of CVD According to Dietary Patterns

The 10-year CVD risk scores were 16.6 ± 0.3, 15.4 ± 0.3, and 15.6 ± 0.2 for men in clusters 1, 2, and 3, respectively (*p* = 0.003). However, the scores were not significantly different in women according to the clusters (Table 2).

### 3.3. Associations between Dietary Patterns and Ten-Year Risk of CVD 

In men, dietary patterns, strength training, and BMI were significant variables associated with the 10-year risk of CVD (R2 = 0.066, Wald F = 4.356, *p* = 0.014). The 10-year risk of CVD was lower in men in cluster 3 (healthy diet) than in those in cluster 1 (typical Korean diet) (t = 2.092, *p* = 0.037). Additionally, the 10-year risk of CVD was lower in men who performed strength training (t = 3.575, *p* < 0.001) than in those who did not. There were no significant differences among women in the three clusters (Table 3).

## 4. Discussion

It is difficult to accurately measure dietary intake, and there are various methods to obtain dietary data and assess dietary patterns [24]. The 24-h dietary recall survey is widely used in the elderly, as it does not require high cognitive ability and can provide qualitative data on the nutrients in foods consumed [24].

In this study, cluster 3 represented participants in the healthy diet group who had a high intake of whole grains, vegetables, fish, and dairy products. This diet was similar to that focused on the intake of unsaturated fatty acids, complex carbohydrates, and dietary fiber recommended for the prevention of CVD [4], Mediterranean diet, and DASH diet [25,26].

The dietary pattern including the intake of white rice is commonly observed in studies of dietary patterns in the Korean elderly, which is similar to the cluster 1 (typical Korean diet) in our study [18,27,28]. The Korean traditional dietary pattern including the intake of a high quantity of white rice, beans, vegetables, kimchi, and seaweed in the elderly over 65 years of age increased from 56.1% in 1998 to 77.6% in 2005 [17], and the Korean diet consumption rate analyzed in the 2007–2009 KNHANES was 80.1% [29]. In this study, 33.7% and 22.6% of men and women, respectively, were included in cluster 1. The Korean diet is recognized as a healthy diet; however, it contains a high amount of carbohydrates and kimchi, which is high in sodium [30].

In this study, the proportion of cluster 3 was high among men and women aged 65–74 years old and those with a high educational level, and high among men high income. In the elderly, educational level [31,32] and income level [32,33] are important factors related to malnutrition. Elderly people with low educational and income levels lack adequate understanding of nutrient intake and the ability to afford adequate food [34]. Thus, they find it difficult to consume adequate nutrients compared to those with high educational and income levels.

The FRS differed depending on sex and men showed poorer lifestyle habits, such as smoking and drinking, than women. Thus, when the FRS was assessed, the risk of developing CVD was higher in men than in women, as variables related to lifestyle habits, such as smoking status, were included. This finding is consistent with those of previous studies that showed differences in the risk of CVD according to sex [35,36].

After adjusting for sociodemographic variables, men in cluster 3 (healthy diet) had a lower 10-year risk of CVD than those in cluster 1 (typical Korean diet). This finding was consistent with that of a study showing that those in the low-risk group for CVD had higher intake of grain, meat, milk, fruit, and vegetables than those in the middle-risk group [37]. Additionally, the result was consistent with those of studies which showed that the DASH diet had beneficial effects on CVD risk indicators [25,38], and those who consumed a Mediterranean diet demonstrated beneficial effects in preventing CVD [39].

In this study, women living alone had a higher 10-year risk of CVD than those living with their spouse. According to Statistics Korea, the proportion of the elderly people living alone gradually increased from 16.0% in 2000 to 23.6% in 2018 [40]. Elderly people living alone were reported to have low health care practices, high stress levels, and poor health status [41]. The elderly may have more health problems due to irregular dietary habits and poor nutritional management.

There are several limitations in this study. First, the KNHANES is a cross-sectional study. Although it assessed the relationship between dietary patterns and CVD risk, the causal relationship could not be clearly defined. Secondly, the 24-h dietary recall survey was useful for identifying individual food and nutrient intakes; however, the survey only assesses the quantity of meals consumed the day before the survey. Therefore, it may be inappropriate in assessing long-term food and nutrient intakes [42]. Third, the Framingham CVD risk score was developed by analyzing non-Asian patients aged 20–79 years. Thus, it may not have been optimal for the participants of this study.

Despite these limitations, this study included a large number of subjects, and the effects of other health behaviors were adjusted. Thus, this study is significant in that it established the type of diet that helps prevent CVD.

## 5. Conclusions

After adjusting for sociodemographic variables, men in cluster 3 (healthy diet) had a lower 10-year risk of CVD than those in cluster 1 (typical Korean diet). When nutrition education programs to improve dietary habits in the elderly are conducted, content on diets consisting of various food groups to prevent CVD is required. In particular, it would be necessary to develop content that emphasizes healthy eating habits in men.

In the nutrition education programs for elderly people in the community, these results can provide them with the information that allows them to choose healthy food for themselves. In addition, when providing food services (meal and side-dish delivery services) in the health care facilities for the elderly or making food as part of self-care activities for the elderly in the community, this information could be an important contribution to cardiovascular disease prevention.

## Figures and Tables

**Table 1:** Participant characteristics classified by dietary pattern cluster.

Characteristics		Male				*p* Value	Female				*p* Value
		Total (*n* = 797)	Cluster 1 (*n* = 275)	Cluster 2 (*n* = 141)	Cluster 3 (*n* = 381)		Total (*n* = 890)	Cluster 1 (*n* = 207)	Cluster 2 (*n* = 276)	Cluster 3 (*n* = 407)	
Age	65–74	527(66.6)	161(29.1)	96(19.0)	270(51.9)	0.005	573(64.9)	104(17.6)	198(35.5)	271(46.9)	<0.001
	≥75	270(33.4)	114(42.9)	45(15.8)	111(41.3)		317(35.1)	103(31.6)	78(22.4)	136(46.0)	
Age (year), mean ± SD		71.9 ± 0.2	73.2 ± 0.3 ^a^	71.2 ± 0.5 ^a^	71.3 ± 0.3 ^b^	<0.001	72.3 ± 0.2	73.9 ± 0.3 ^a^	71.4 ± 0.3 ^ab^	72.1 ± 0.3 ^b^	<0.001
Marital status	Living alone	100(12.1)	49(44.7)	17(13.3)	34(42.0)	0.063	426(49.0)	112(25.4)	122(29.2)	192(45.5)	0.223
	Living together	697(87.9)	226(32.2)	124(18.6)	347(49.2)		464(51.0)	95(19.9)	154(32.5)	215(47.6)	
Education	≤Elementary school	340(41.8)	161(49.3)	59(17.1)	120(33.6)	<0.001	705(77.9)	198(27.8)	205(28.3)	302(43.8)	<0.001
	Middle school	142(17.6)	51(33.3)	26(19.0)	65(47.7)		87(9.6)	8(7.7)	28(32.9)	51(59.4)	
	High school	184(23.0)	51(25.4)	36(19.6)	97(55.0)		75(9.5)	1(1.4)	32(41.9)	42(56.7)	
	≥College	131(17.5)	12(8.0)	20(16.6)	99(75.4)		23(3.0)	0(0.0)	11(56.5)	12(43.5)	
Income	Low (quartile 1)	200(25.7)	91(45.3)	37(18.6)	72(36.1)	0.001	219(24.6)	69(27.3)	56(28.6)	94(44.1)	0.166
	Low-medium (quartile 2)	195(23.5)	75(36.8)	32(17.7)	88(45.5)		244(26.0)	64(26.8)	72(29.9)	108(43.3)	
	High-medium (quartile 3)	193(22.0)	60(29.5)	40(21.6)	93(48.9)		216(23.8)	42(20.0)	72(33.0)	102(47.0)	
	High (quartile 4)	209(28.8)	49(24.1)	32(14.7)	128(61.2)		211(25.6)	32(16.2)	76(32.0)	103(51.8)	
Strength training	<2 per week	580(71.0)	213(35.7)	102(18.2)	265(46.1)	0.198	817(91.7)	198(23.7)	252(30.4)	367(45.8)	0.038
	≥2 days per week	217(29.0)	62(28.9)	39(17.3)	116(53.8)		73(8.3)	9(9.4)	24(36.1)	40(54.4)	
Smoking status	Current smoking	641(79.9)	211(32.4)	114(18.4)	316(49.3)	0.350	871(97.3)	200(22.3)	271(31.0)	400(46.6)	0.761
	Non/past smoking	156(20.1)	64(39.2)	27(16.2)	65(44.5)		19(2.7)	7(30.2)	5(26.3)	7(43.5)	
High-risk drinking	No	734(92.7)	257(34.3)	115(15.8)	362(49.9)	<0.001	882(99.2)	206(22.7)	274(31.0)	402(46.3)	0.183
	Yes	63(7.3)	18(26.4)	26(45.4)	19(28.2)		8(0.8)	1(8.2)	2(16.8)	5(75.0)	
Hypertension	No	433(53.8)	139(31.1)	74(16.6)	220(52.3)	0.113	410(45.6)	88(20.8)	128(31.3)	194(47.9)	0.613
	Yes	364(46.2)	136(36.8)	67(19.5)	161(43.7)		480(54.4)	119(24.0)	148(30.6)	213(45.4)	
Diabetes mellitus	No	687(87.2)	239(33.9)	121(17.6)	327(48.5)	0.875	762(84.1)	178(21.9)	241(31.7)	343(46.4)	0.548
	Yes	110(12.8)	36(32.7)	20(19.9)	54(47.4)		128(15.9)	29(25.9)	35(26.5)	64(47.6)	
Dyslipidemia	No	689(85.8)	248(35.6)	123(18.0)	318(46.3)	0.038	603(66.9)	161(26.3)	166(27.4)	276(46.2)	0.001
	Yes	108(14.2)	27(22.3)	18(17.3)	63(60.4)		287(33.1)	46(14.9)	110(37.9)	131(47.2)	
Body mass index (m^2^/kg), mean ± SD		23.5 ± 0.1	23.5 ± 0.2	23.7 ± 0.3	23.4 ± 0.2	0.738	24.2 ± 0.1	24.3 ± 0.3	24.1 ± 0.2	24.1 ± 0.2	0.826

Values are presented as numbers (%) or mean ± SD. *p* values were determined by Chi-squared test and one-way analysis of variance (ANOVA). The significant difference between the groups indicated by superscript letters (Bonferroni’s post hoc test) a = *p* < 0.05 vs. cluster 3; b = *p* < 0.05 vs. cluster 1.

**Table 2:** Ten-year risk for cardiovascular disease (CVD) according to dietary patterns.

Dietary Patterns		Ten-Year Risk for CVD (Total)	*p* Value
Male	Cluster 1	16.6 ± 0.3 ^a^	0.003
	Cluster 2	15.4 ± 0.3 ^a^	
	Cluster 3	15.6 ± 0.2 ^b^	
	Total	15.9 ± 0.2	
Female	Cluster 1	13.5 ± 0.4	0.099
	Cluster 2	12.6 ± 0.2	
	Cluster 3	13.0 ± 0.2	
	Total	13.0 ± 0.2	

Values are presented as mean ± SD. *p* values were determined by ANOVA. The significant difference between the groups indicated by superscript letters (Bonferroni’s post hoc test) a = *p* < 0.05 vs. cluster 3; b = *p* < 0.05 vs. cluster 1. Cluster1; typical Korean diet, Cluster2; high carbohydrate diet, Cluster3; healthy diet. CVD; cardiovascular disease.

**Table 3:** Factors which influence total ten-year CVD risk.

Factors	Male					Female				
	B	95.0% CI for B		t	*p* Value	B	95.0% CI for B		t	*p* Value
		Lower	Upper				Lower	Upper		
Dietary pattern (reference: cluster 3)										
Cluster 1	0.721	0.043	1.398	2.092	0.037	0.264	−0.604	1.132	0.597	0.551
Cluster 2	−0.437	−1.104	0.230	−1.288	0.199	−0.286	−0.925	0.353	−0.880	0.379
Marital status (reference: living together)										
Living alone	0.645	−0.242	1.533	1.431	0.153	1.264	0.706	1.823	4.451	<0.001
Education (reference: ≥ College)										
≤ Elementary school	0.766	−0.107	1.640	1.726	0.085	1.449	−0.320	3.217	1.611	0.108
Middle school	0.137	−0.752	1.026	0.303	0.762	1.408	−0.526	3.343	1.432	0.153
High school	0.659	−0.207	1.525	1.496	0.136	0.137	−1.746	2.020	0.143	0.886
Income (reference: High (quartile 4))										
Low (quartile 1)	0.094	−0.747	0.935	0.219	0.827	−0.063	−0.869	0.743	−0.153	0.878
Low-medium (quartile 2)	−0.130	−0.943	0.683	−0.315	0.753	−0.349	−1.123	0.426	−0.886	0.376
High-medium (quartile 3)	0.115	−0.709	0.939	0.275	0.783	−0.570	−1.352	0.213	−1.432	0.153
High-risk drinking (reference: Yes)										
No	−0.689	−1.607	0.229	−1.477	0.141	−1.645	−4.089	0.798	−1.324	0.186
Strength training (reference: No)										
Yes	−1.109	−1.720	−0.499	−3.575	<0.001	−0.017	−1.072	1.038	−0.032	0.974
Body mass index (m^2^/kg)	0.096	0.010	0.183	2.194	0.029	0.166	0.081	0.250	3.851	<0.001
	R2 = 0.066, Wald F (*p*) = 4.356 (0.014)					R2 = 0.078, Wald F (*p*) = 0.893 (0.410)				

CI: confidence interval. *p* values were determined by complex sample multiple linear regression analysis. Cluster1; typical Korean diet, Cluster2; high carbohydrate diet, Cluster3; healthy diet. Variable included in the ten-year CVD risk score (age, smoking status, Diabetes mellitus, Hypertension, total cholesterol) were excluded.

## Data Availability

Publicly available datasets were analyzed in this study. This data can be found here: https://knhanes.cdc.go.kr/knhanes/main.do, accessed on 26 November 2019.

## Supplementary Materials

The following are available online at https://www.mdpi.com/article/10.3390/ijerph18073703/s1, Table S1: Food groups and items used in the dietary pattern analysis; Table S2: consumption of food groups (g/day or mL/day^§^) by cluster, *n* = 1687.
