# Supplementary material for: Relationship between Dietary Patterns and Cardiovascular Disease Risk in Korean Older Adults
Source: Int J Environ Res Public Health. 2021 Apr 1;18(7):3703. doi: 10.3390/ijerph18073703 (PMC8038041; doi:10.3390/ijerph18073703)
Supplement: Supplementary file 1 [file ijerph-18-03703-s001.pdf]

Table S1. Food groups and items used in the dietary pattern analysis.

Food Group	Food Items
White rice	White rice
Other grain	Brown rice, barley, millet, maize, etc.
Noodles and dumpling	Noodle, ramen, somyon, udong, dumpling, etc.
Flour and bread	Wheat flour, breads, doughnuts, cakes, etc.
Pizza, hamburger, cereals and snack	Pizza, hamburger, sandwich, cereal, cracker, snack, cookie, popcorn, biscuits, chip, etc.
Potatoes	Potato, french fried potato, sweet potato, etc.
Sugars and sweets	White sugar, honey, candy, chocolates, jelly, caramel, etc.
Legumes	Soybean, curd, green peas, red bean, etc.
Nuts and seeds	Peanuts, almonds, walnut, sunflower seed, etc.
Vegetables	All kinds of vegetables, etc.
Kimchi	All kinds of kimchi, etc.
Mushrooms	All kinds of mushroom, etc.
Fruits	Citrus fruit, strawberry, melon, banana, peach, orange, fruit juices, etc.
Meats and meat products	Beef, pork, ham, sausage, meatball, bacon, chicken, turkey, etc.
Eggs	Egg, cooked egg, fried egg, etc.
Fishes and seafood	Mackerel, bastard halibut, flat fish, canned tuna, anchovy, squid, etc.
Seaweeds	Seaweed, kelp, etc.
Milk and dairy products	Milk, ice cream, yoghurt, cheese, etc.
Oils	Olive oil, corn oil, sesame oil, etc.
Beverages and alcohol	Coffee, green tea, black tea, hot chocolate, carbonated beverage, beer, liquor, whiskey, etc.
Seasonings	Soy sauce, red pepper, mayonnaise, ketchup, salt, etc.
Etc.	Etc.

Table S2. Consumption of food groups (g/day or ml/day§) by cluster, n = 1,687.

Food Group	Male			Female		
	**Cluster 1 (n = 275)**	**Cluster 2 (n = 141)**	**Cluster 3 (n = 381)**	**Cluster 1 (n = 207)**	**Cluster 2 (n = 276)**	**Cluster 3 (n = 407)**
%	33.7	17.9	48.3	22.6	30.9	46.6
White rice	29.4	12.7	12.7	39.6	15.8	13.9
Other grain	3.3	3.3	3.5	3.8	2.8	4.6
Noodles and dumpling	2.4	2.5	2.8	1.0	1.3	2.6
Flour and bread	0.8	1.2	1.1	0.8	1.1	1.2
Pizza, hamburger, cereals and snack	0.2	0.3	0.2	0.1	0.2	0.3
Potatoes	1.8	1.8	3.4	1.8	2.5	4.4
Sugars and sweets	0.9	0.6	0.8	0.5	0.5	0.6
Legumes	4.5	3.0	3.2	3.7	2.6	3.8
Nuts and seeds	0.5	0.4	0.5	0.4	0.3	0.3
Vegetables	15.3	12.7	16.4	13.3	11.9	20.4
Kimchi	17.0	8.0	7.7	15.9	8.4	7.4
Mushrooms	0.2	0.2	0.3	0.4	0.2	0.3
Fruits	3.5	4.3	20.9	4.2	35.6	8.2
Meats and meat products	5.3	4.3	3.9	2.3	2.8	3.5
Eggs	1.2	1.5	1.4	0.7	1.1	1.4
Fishes and seafood	3.4	5.5	8.3	3.0	3.4	9.7
Seaweeds	0.7	1.1	3.2	0.5	1.1	3.8
Milk and dairy products§	2.3	2.3	3.9	2.6	4.1	5.5
Oils§	0.4	0.4	0.4	0.3	0.3	0.4
Beverages and alcohol§	4.2	31.4	2.7	2.2	2.4	5.5
Seasonings§	2.3	1.8	1.9	2.5	1.4	2.0
Etc.	0.4	0.5	0.8	0.3	0.3	0.3
